# Ion Conduction Dynamics, Characterization, and Application of Ionic Liquid Tributyl Methyl Phosphonium Iodide (TMPI)-Doped Polyethylene Oxide Polymer Electrolyte

**DOI:** 10.3390/polym17141986

**Published:** 2025-07-19

**Authors:** Suneyana Rawat, Monika Michalska, Pramod K. Singh, Karol Strzałkowski, Nisha Pal, Markus Diantoro, Diksha Singh, Ram Chandra Singh

**Affiliations:** 1 Centre of Excellence in Solar Cells & Renewable Energy, Department of Physics and Environmental Science, SSES, Sharda University, Greater Noida 201310, India; nishapal.sharda@gmail.com (N.P.); rcsingh@sharda.ac.in (R.C.S.); 2 Department of Chemistry and Physico-Chemical Processes, Faculty of Materials Science and Technology, VŠB-Technical University of Ostrava, 17. listopadu 2172/15, 708 00 Ostrava-Poruba, Czech Republic; 3 Institute of Physics, Faculty of Physics, Astronomy and Informatics, Nicolaus Copernicus University, Grudziadzka 5, 87-100 Torun, Poland; skaroll@fizyka.umk.pl (K.S.); diksha@doktorant.umk.pl (D.S.); 4 Department of Physics, Faculty of Mathematics and Natural Science, Universitas Negeri Malang, Semarang 5, Malang 65145, Indonesia; markus.diantoro.fmipa@um.ac.id

**Keywords:** electrolyte, ionic liquids, DSSC, EDLC, storage devices

## Abstract

The increasing demand for high-performance energy storage devices has stimulated interest in advanced electrolyte materials. Among them, ionic liquids (ILs) stand out for their thermal stability, wide electrochemical windows, and good ionic conductivity. When doped into polymeric matrices, these ionic liquids form hybrid polymeric electrolytes that synergize the benefits of both liquid and solid electrolytes. This study explores a polymeric electrolyte based on polyethylene oxide (PEO) doped with tributylmethylphosphonium iodide (TMPI) and ammonium iodide ($NH_{4}I$), focusing on its synthesis, structural and electrical properties, and performance in energy storage devices such as dye-sensitized solar cells and supercapacitors. Strategies to improve its ionic conductivity, mechanical and chemical stability, and electrode compatibility are also discussed, along with future directions in this field.

## 1. Introduction

The global transition to renewable energy technologies requires the development of efficient and sustainable energy storage solutions. Among the various energy storage technologies, lithium-ion batteries and supercapacitors are dominant due to their energy density and power capabilities. A critical component of these devices is the electrolyte, which facilitates ion transport between electrodes while maintaining electrical insulation to prevent short circuits. Conventional electrolytes, particularly organic solvent-based liquid electrolytes, offer high ionic conductivity but are associated with significant safety concerns. These include flammability, toxicity, and a propensity to leak or evaporate, making them less desirable for long-term or large-scale applications [1].

Ionic liquids (ILs) offer an attractive alternative to traditional electrolytes. The origin of ionic liquids (ILs) dates back to an observation by Paul Walden in 1914, where he documented the characteristics of ethylammonium nitrate, which is considered to be the first known IL [2]. The contemporary definition of ILs remains based on his work, characterizing them primarily as salts that exist in a liquid state at relatively low temperatures (often below 100 °C). Ionic liquids are composed entirely of ions: positively charged cations and negatively charged anions [3]. This ionic nature imparts several desirable properties, such as low volatility, high thermal stability, and a wide electrochemical stability window, making ILs attractive for next-generation applications. Ionic liquids have received great attention from researchers due to their potential applications, with excellent chemical and physical properties. Ionic liquids have a wide range of properties, such as non-volatility, a wide electrochemical window, and high thermal stability [4]. With these successful properties, ionic liquids are considered some of the best material choices for lithium energy storage fabricated using advanced technology in the world. Organic anions and inorganic cations that are poorly bonded are known as ionic liquids. This asymmetric structure makes it difficult for them to crystallize. Therefore, they remain liquid in nature within a very wide temperature range [5].

For the first generation of ionic liquids, attention has been paid to imidazolium-based cations due to their high conductivity and very low viscosity [6]. But in view of their low electrochemical windows, as acidic protons have, they have limited applications. Phosphonium ionic liquids have the particularly powerful properties of low vapor pressure, a high heat capacity, and wide-range liquidity [7]. Ionic liquid based on a phosphonium architecture has specific properties compared to those of its ammonium, pyridinium, and imidazolium cation counterparts, as it has no acidic proton. Phosphonium-based ionic liquids are a specialized category within the broader family of ionic liquids. The defining feature of phosphonium-based ionic liquids is their phosphonium cation, which is built around a phosphorus atom bonded to four organic substituents (alkyl or aryl groups). This structure distinguishes them from other ionic liquids that commonly use ammonium, imidazolium, or pyridinium cations [8]. Phosphonium cations have a fourth different substitution and offer a very good opportunity to bind with the greatest number of anions. Phosphonium-based ionic liquids exhibit a larger thermal stability window. Their higher viscosity restricts the mobility of ionic liquids and offers very low conductivity compared to that of other ionic liquids. The cation–anion interaction results in high viscosity and higher delocalization of the cations composing the ionic liquid at room temperature [9].

Phosphonium-based ionic liquids have emerged as promising additives for polymer electrolytes. These ionic liquids, composed of phosphonium cations paired with various anions, exhibit unique physicochemical properties, such as low volatility, high thermal stability, and wide electrochemical stability windows. By doping phosphonium-based ionic liquids into polymer matrices, researchers aim to combine the mechanical robustness of polymers with the superior ionic conductivity of ionic liquids [10].

A significant example of a phosphonium-based ionic liquid is tributylmethylphosphonium iodide (TMPI). Tributylmethylphosphonium iodide (TMPI) is an ionic liquid that is characterized by a substantial phosphonium cation and a relatively small iodide anion with the molecular formula $\left(C_{13}H_{30}IP\right)$, and this specific composition is responsible for its distinctive properties, including low volatility, high thermal stability, and superior solvation capabilities. In this study, we show that the combination of TMPI ionic liquid with polyethylene oxide (PEO) and saline ammonium iodide ($NH_{4}I$) resulted in a free-standing and easy-to-handle polymer film that had high conductivity at room temperature. These polymer films are also used for high-efficiency energy storage devices.

## 2. The Experimental Procedure

### 2.1. Polymer Electrolyte Preparation

First, high-molecular-weight polyethylene—PEO ($Mw\;=\;10^{5}$, Sigma Aldrich (St. Louis, MO, USA)) —and 8 wt.% ammonium iodide—$NH_{4}I$ (Sigma Aldrich)—were mixed together to form a polymeric solution, as already published, and stirred for 4–5 h until homogeneous [11]. Different compositions of the ionic liquid, TMPI, ranging from 2 wt.% to 24 wt.% were added to the polymer–salt complex and magnetically stirred at room temperature overnight until complete dissolution. The ionic-liquid-doped solution were subsequently cast into different Petri dishes and permitted to evaporate gradually at room temperature.

### 2.2. Dye-Sensitized Solar Cell Fabrication

DSSCs utilizing the TMPI-doped polymer electrolyte were constructed in a configuration of FTO glass/TiO_2_/N3-dye/TMPI/polymer electrolyte-I_2_/H_2_PtCl_6_/FTO glass by interposing the polymer electrolyte between the dye-adsorbed TiO_2_ electrode and the chloroplatinic-acid-coated FTO, as already reported [12]. The photocurrent–voltage (J-V) characteristics of the cell were evaluated under a solar simulator under one sun illumination of 100 mWcm^−2^.

### 2.3. Electric Double-Layer Capacitor Fabrication

The plastic-waste-based activated carbon underwent drying for an entire night at approximately 100 °C prior to its application. The electrode was formulated by combining activated carbon, carbon black (Alfa Aesar), and PVDF-HFP as the binder in alcohol at a weight ratio of 8:1:1. This slurry was then applied onto a flexible graphite sheet (with a 0.5 mm thickness). Subsequently, the activated carbon electrodes were placed in an oven and dried at 50 °C for 1–2 h. The fabrication of the EDLC cell involved sandwiching TMPI-doped polymer electrolytes between two activated carbon electrodes with a $1\times 1\;cm^{2}$ area.

## 3. Characterization and Results

### 3.1. The Polarized Optical Microscope

**Figure 1:**
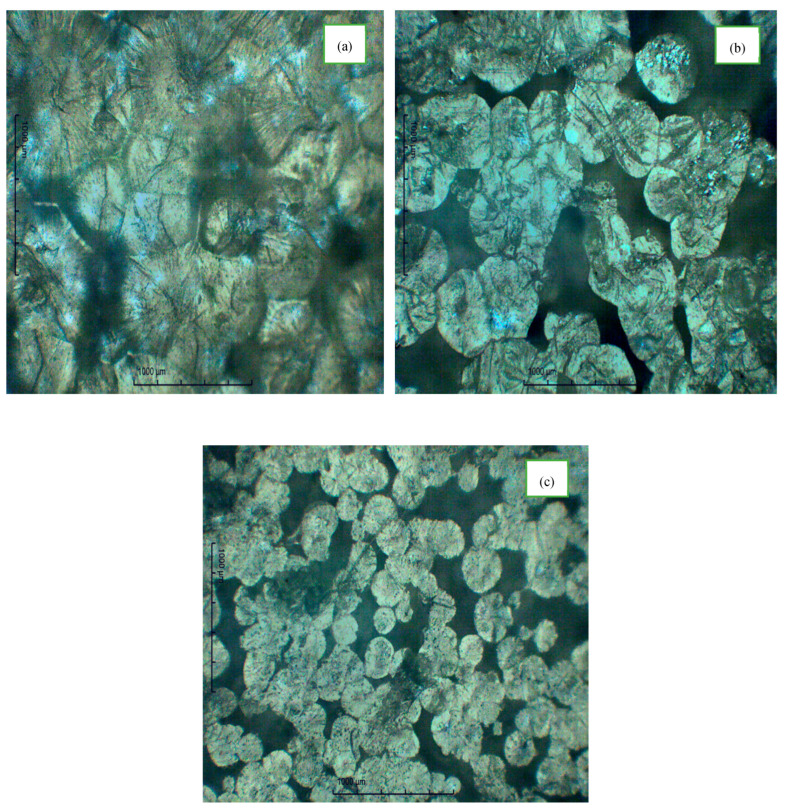
Polarized optical micrograph of (**a**) undoped *PEO*, (**b**) *NH*_4_*I* complex with *PEO*, and (**c**) *TMPI*-doped *PEO*: *NH*_4_*I* polymer electrolyte.

The optical micrographs presented in Figure 1 illustrate the characterization of pure PEO, the PE complex with ammonium iodide, and $PEO:NH_{4}I\;$ incorporated with IL–TMPI. The characteristic spherulitic texture of PEO is evident and reveals its lamellar crystalline configuration, with dark boundaries signifying the presence of an amorphous region within the polymeric matrix (Figure 1a) [13]. Upon the formation of the PEO complexes with $NH_{4}I$, a remarkable alternation in the spherulitic pattern is observed, accompanied by the increased presence of boundaries and dark regions (Figure 1b) [12]. This suggests a significant increase in the amorphous content, leading to a decrease in the crystallinity of the materials. The decrease in crystallinity of the polymeric complex was also validated by the DSC results, which will be explained in the next section. A significant change in the spherulitic arrangement, and thus in the crystalline properties, was observed upon the incorporation of the ionic liquid–TMPI into the PEO complex (Figure 1c). It was observed that the addition of IL–TMPI to the polymer–salt complex resulted in a progressive increase in the amorphous region (dark region). The PEO:$\;NH_{4}I$:TMPI polymeric electrolyte matrix contributes to an increased level of amorphicity, characterized by a remarkable expansion of the dark and densely packed area, together with a significant reduction in the size of the spherulites, as illustrated in Figure 1c [14].

The crystalline domains within the polymer matrix of the polymer electrolyte impede ionic conduction due to the exclusion of electrolyte salts from these densely arrange polymer chain regions. Each of these crystallites in the polymer matrix serves as a barrier to the diffusion of salt and affects the ion conduction. The incorporation of $NH_{4}I$ and TMPI disrupts the crystalline domain of PEO, increasing the amorphous region within the polymer matrix. As already reported in the literature, a greater amorphous region in a polymer electrolyte is related to higher ionic conductivity [15]. The amorphous phase facilitates improved ion percolation, thereby improving ion mobility and transport rates.

### 3.2. Fourier Transform Infrared Spectroscopy

**Figure 2:**
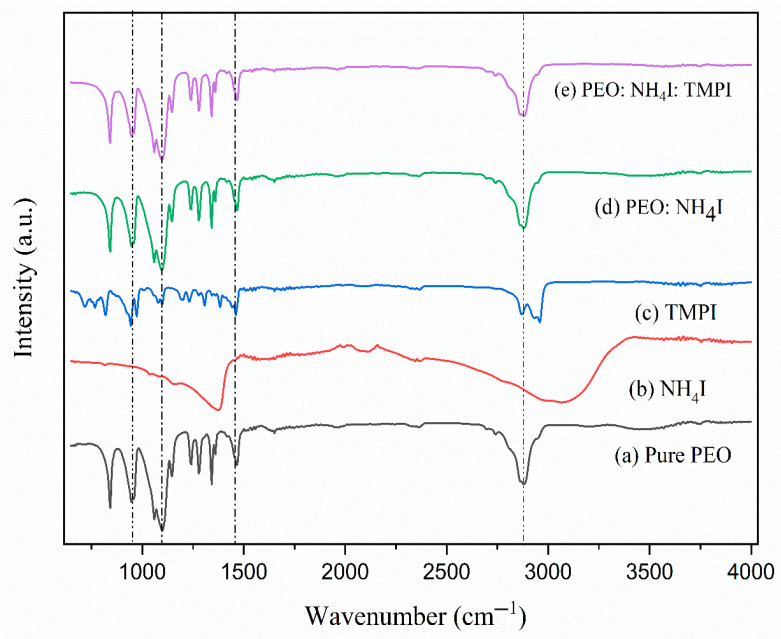
FTIR peaks observed for (a) pure PEO, (b) $NH_{4}I$, (c) TMPI, (d) $PEO:NH_{4}I$ polymer electrolyte, and (e) $PEO:NH_{4}I:TMPI.$

The Fourier transform infrared (FTIR) spectra for undoped PEO, ammonium iodide ($NH_{4}I$), IL–TMPI, the PEO complex with the $NH_{4}I$ polymer electrolyte, and the IL–TMPI-infused $PEO:NH_{4}I$ polymer electrolyte are illustrated in Figure 2, with the associated peak assignments detailed in Table 1.

In the spectrum of the pure PEO, peaks corresponding to its polymeric arrangement are exhibited. The preeminent peak appears at $1092\;cm^{-1},$ as shown in Figure 2, attributed to the C-O-C stretching vibration of the ether linkage within the polymer matrix backbone, as mentioned in Table 1 [16]. Further, the peak at $1465\;cm^{-1}$ corresponds to C–H bending, indicating the presence of a methylene group in the matrix. The C–H wagging peak at $1342\;cm^{-1}$ and the C–H  stretching peak at $2878\;cm^{-1}$ represent the bending vibrations of C–H bonds and symmetric/asymmetric stretching of the methylene group [17]. Meanwhile, pure $NH_{4}I$ exhibits a broad spectrum at $3340\;cm^{-1}$, which indicates the presence of N–H stretching due to symmetric and asymmetric vibrations. This broadening is primarily due to the presence of hydrogen bonding between the ${NH}_{4}^{+}$ ions [18]. On the other hand, the ionic liquid–TMPI shows the characteristic peaks at $812\;cm^{-1}$ associated with the phosphonium ring of the phosphonium cation, while the $939\;cm^{-1}$ peak is associated with P–I stretching, which represents the phosphonium and iodide interaction. $P–CH_{3}$ symmetric stretching corresponds to the phosphonium ether group at the $1384\;{\mathrm{cm}}^{-1}$ peak [19].

**Table 1:** FTIR peak positions with corresponding assignments.

Material	$\mathrm{Vibrational}\;\mathrm{Frequency}\;({\mathrm{cm}}^{-1})$	Band Assignment	Class	Ref.
PEO	842	$CH_{2\;\;}$ rocking	$\mathrm{Methylene}\;–CH_{2\;\;}–$	[20]
	956	Crystalline phase	-	[21]
	1092.93	Anti-symmetric bridge C–O–C stretching	Ether	[22]
	1342.03	C–H wagging	$\mathrm{Methylene}\;–CH_{2\;\;}–$	[23]
	1465.12	C–H bending	$\mathrm{Methylene}\;–CH_{2\;\;}–$	[23]
	2878.07	C–H stretching	$\mathrm{Methylene}\;–CH_{2\;\;}–$	[23]
$NH_{4}I$	1372	N–H bending	$\mathrm{Deformation}\;\mathrm{of}\;NH_{4}^{-}$	[24]
	3066	N–H stretching	Symmetric/asymmetric N-H bond	[24]
TMPI	435	P–I interaction	Phosphonium–iodide interaction	[25]
	812	Phosphonium ring	Phosphonium	[26]
	939	P–I stretching	Phosphonium– iodide	[27]
	1096	I–P interaction	Phosphonium– iodide	[28]
	1386	$P–CH_{3\;\;}$ symmetric stretching	Phosphonium ether group	[28]
	1466	C–H bending	$\mathrm{Alkyl}\;\mathrm{groups}\;–CH_{2}–\;or\;–CH_{3}–$	[29]
	2870	C–H stretching	$\mathrm{Methylene}\;–CH_{2\;\;}–$	[29]
	2957	C–H stretching	$\mathrm{Methylene}\;–CH_{2\;\;}–$	[29]
$PEO:NH_{4}I$	634	$I^{-\;}$ vibration	$\mathrm{Lattice}\;\mathrm{vibration}\;(I^{-})$	[30]
	1135	Anti-symmetric bridge C–O–C stretching	Ether	[31]
	1280	C–H wagging	$\mathrm{Bending}\;\mathrm{vibration}\;CH_{2\;\;}$	[12]
	2857	C–H stretching	$\mathrm{Methylene}\;–CH_{2\;\;}–$	[31]
	3329	N–H stretching	Symmetric/asymmetric N-H bond	[31]
$PEO:NH_{4}I:TMPI$	740	$P–CH_{2}$ rocking	Phosphonium group	[32]
	1031	P–CH stretching	Phosphonium group	[33]
	1130	Anti-symmetric bridge C–O–C stretching	Ether	[20]
	1280	C–H wagging	$\mathrm{Methylene}\;–CH_{2\;\;}–$	[34]
	2870	C–H symmetric stretching	$\mathrm{Methylene}\;–CH_{2\;\;}–$ of TMPI	[30]
	2879	C–H stretching	PEO	[23]
	3340	Symmetric/asymmetric N–H stretching	$NH_{4}I$	[12]

In Figure 2, curve (d), the FTIR spectrum of the salt–NH4I-infused PEO polymer electrolyte is revealed, which exhibits characteristic peaks of both components and incidentally no structural modification. At 1135$\;cm^{-1}$, the peak associated with C–O–C stretching remains the same, suggesting very minimal interaction at this site. There is an intense checkerboard peak at 3329 $cm^{-1}$ due to N–H stretching that appears to reflect the interaction between the PEO chain and the ${NH}_{4}^{+}$ ion. This observation reveals that the interaction between the polymer and the salt is ionic rather than covalent [35]. Furthermore, with the incorporation of the IL-TMPI into the PEO:NH_4_I complex, as shown in Figure 2, curve (e), there is a slight shift in the peak at $1130\;cm^{-1}$ of C–O–C stretching to a lower wavelength, indicating complexation between the phosphonium cation from TMPI and the PEO matrix. A broadening effect, accompanied by a slight redshift, is observed due to the N–H stretching peak detected at $3340\;cm^{-1}$. This phenomenon suggests a remarkable enhancement in the hydrogen bonding interaction within the polymer matrix [36]. Moreover, specific peaks associated with the presence of the ionic liquid are prominently observed in the C–H asymmetric peak at $2879\;cm^{-1}$ and the C–H symmetric peak at $2870\;cm^{-1}$. The clear observation of these peaks provides strong evidence for the successful integration of TMPI into the PEO matrix. Other peaks, such as $P–CH_{2}$ rocking and P–CH wagging at $740\;cm^{-1}$ and $1130\;cm^{-1}$, also confirmed the presence of the phosphonium cation in the polymer electrolyte [37].

### 3.3. Scanning Calorimetry

**Figure 3:**
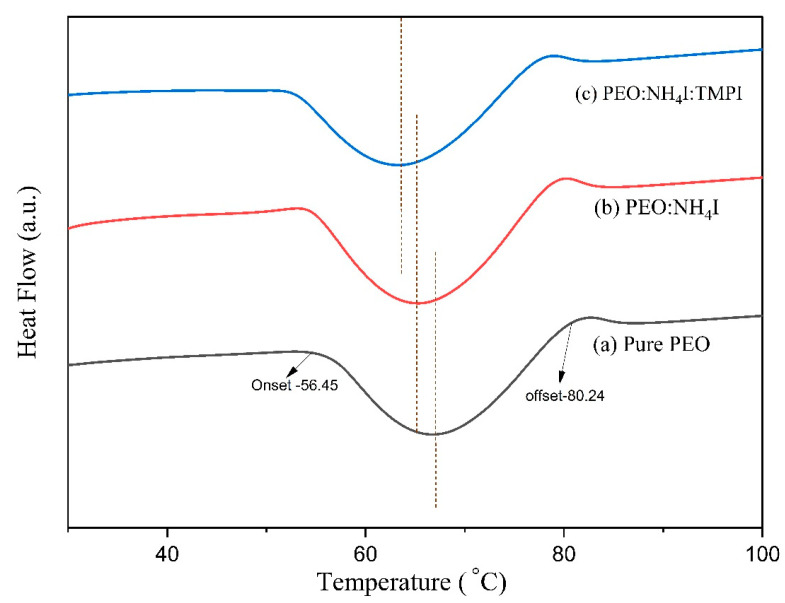
DSC thermograms of (a) pure PEO, (b) PEO:NH_4_I, and (c) PEO:NH_4_I:TMPI in the range of 20–100°C.

Figure 3 illustrates the exothermic DSC thermograms for the pure PEO, $PEO:NH_{4}I$, and $PEO:NH_{4}I:TMPI$ polymer electrolytes in the range of 20–100 °C (see curves ‘a’, ‘b’, and ‘c’ in Figure 3), with detailed data provided in the subsequent table. Based on the DSC analysis, the melting range was calculated using Equation (1) since this is a critical parameter for assessing the quality and perfection of the crystalline structure within the materials.

(1) $${∆T}_{m}=T_{offset}\;-\;T_{onset}$$

where ${∆T}_{m}$ is denoted as the melting range, $T_{offset}$ is the offset temperature, and $T_{onset}$ is the onset temperature. The pure PEO polymer electrolyte has a melting range of ΔT_m_ = 23.77 °C calculated from the onset and offset temperatures, i.e., 56.45 °C and 80.23 °C, using Equation (1), indicating the presence of semi-crystalline regions with varying perfection in the crystal configuration. As already reported, the melting range exhibits an inverse relationship with the degree of perfection of crystallization, signifying that a narrower melting range is due to a well-ordered and highly crystalline material, as shown for pure PEO [38]. Further using the theoretical value of enthalpy of fusion for perfectly crystalline PEO, i.e., 213.7 J/g [39], the degree of crystallinity was evaluated as 72.74% using Equation, which confirmed the highly crystalline fraction within the pure PEO.

(2) $$\chi_{c}=\frac{{∆H}_{m}}{{∆H}_{0}}\times 100$$

where $\chi_{c}$ is the degree of crystallinity, ${∆H}_{0}$ indicates the standard enthalpy of fusion of pure PEO, and ${∆H}_{m}$ refers to the enthalpy of fusion associated with the synthesized PEO polymer electrolyte.

Upon the addition of salt, the regular packing of the PEO chains is disrupted as the degree of crystallinity decreases to 56.37, as shown in the Table 2. This is due to the ionic interaction between the ammonium ion from the salt and the oxygen present in the ether of PEO, which interferes with chain crystallization and increases the amorphous content, as it functions as a defect-inducing agent. Meanwhile, the incorporation of the ionic liquid decreases the χc value to 33.67, thereby reducing the chain-to-chain interactions of the polymer [40]. As TMPI acts as a plasticizer when added to the polymer matrix, the free volume and chain mobility increase. This is due to the strong interactions between the phosphonium cation and the polymer chains that weaken the crystallinity further, increasing the amorphous region in the polymer electrolyte, as is also mapped in the XRD data.

Despite the observed decrease in crystallinity, the melting range exhibits remarkable stability, remaining at a value of approximately 23 °C. ΔT_m_ is primarily affected by the quality and spatial distribution of the crystallites present in the polymer. In each of the three compositions, the variation in the crystallite size remains wide and comparable. However, it is important to note that the overall degree of crystallinity shows a downward trend. This reduction in crystallinity can have implications for the thermal properties of the polymer electrolyte, potentially affecting its melting behavior and stability [41].

### 3.4. The Thermogravimetric Analysis

**Figure 4:**
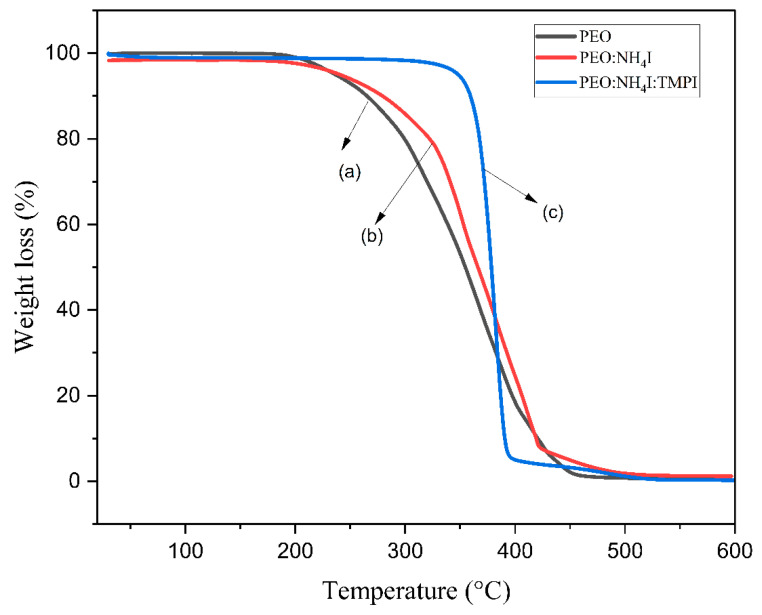
TGA thermograms of (a) pure PEO, (b) PEO:NH_4_I, and (c) TMPI-doped PEO:NH_4_I polymer electrolyte.

Thermogravimetric analysis (TGA) is an essential method for evaluating the thermal stability of synthesized polymer electrolytes. This work presents the TGA profiles for three different materials, pure PEO, the PEO:NH_4_I complex, and TMPI-IL-doped PEO, which are illustrated in Figure 4. The TGA was performed over a wide temperature range, from 30 °C to 600 °C. The thermograms obtained for the pure PEO polymer electrolyte film demonstrate a one-step decomposition process; initially, the electrolyte is stable up to 100 °C due to the evaporation of moisture and other volatile substances; from 150 to 450 °C, a steep decline appears with the onset of decomposition at approximately 276.39 °C, as shown in Figure 4, curves a, which is related to the decomposition of the polymer backbone. When ammonium iodide (NH_4_I) is added to the polymer matrix, the onset of thermal decomposition shifts slightly toward the lower temperature. Since the salt is hygroscopic in nature, this contributes to the remaining stable inorganic residues after the complete decomposition of the polymer’s molecular structure [42].

The incorporation of the TMPI ionic liquid shows a significant change in the TGA plot, as it displays two-step decomposition, as shown in Figure 4, curve (c). Initially thermally stable below 300 °C with minimal weight loss, the first-step decomposition reveals significant weight loss that starts to occur at an initial thermal temperature of 355.31 °C, representing the complete breakdown of the polyethylene’s molecular structure. The second-step decomposition takes place between 400 and 500 °C, which is due to the degradation of the ionic liquid, which is much higher than that for the PEO:NH_4_I complex due to the strong interaction between the ionic liquid and the polymer. The TGA thermograms for the TMPI-doped polymer:salt complex show thermal stability at higher temperatures. This step involves the degradation of both the phosphonium cations and the iodide anions of TMPI, culminating in the final weight loss. This two-stage decomposition confirms that the ionic liquid not only modified the thermal decomposition pathways but also improved the overall stability of the polymer electrolyte [43]. TGA thermograms of the TMPI-doped polymer–salt complex show its thermal stability at higher temperatures.

### 3.5. Complex Impedance Spectroscopy

In this study, we focused on the development of solid polymer electrolytes by incorporating phosphonium-based ionic liquid, which acts as a plasticizer, into a polymer matrix. An important aspect of this research was evaluating how these interactions contributed to the enhancement of the polymer electrolyte conductivity by the ionic liquid. The ionic conductivities of the synthesized polymer electrolytes were evaluated using an electrochemical impedance spectroscopy workstation. Measurements were made across a frequency spectrum of $10^{2}$ Hz to $10^{6\;}$ Hz at room temperature. The sample was placed between custom-made stainless steel ion-blocking electrodes, which included working and counter (auxiliary) electrodes. A low-amplitude alternating current of 10 mV was introduced across the electrodes, and the resulting current was subsequently recorded. The bulk resistance (R_b_) was identified in the low-frequency area of the Nyquist plot, and the ionic conductivity of the polymer electrolyte could be determined using Equation (3).

(3) $$\sigma =\frac{l}{R_{b}A}$$

where σ is the ionic conductivity (S/cm), l is the thickness of the synthesized polymer electrolyte, and A is the tested area of the polymer electrolyte. Previous reports indicate that pure PEO demonstrates conductivity peaks at $2.96\times 10^{-8}\;S/cm.\;$ On adding NH_4_I into the polymer matrix, the ionic conductivity increases to the value of $2.05\times 10^{-6}\frac{S}{cm},\;$ as already reported in the literature [12].

**Figure 5:**
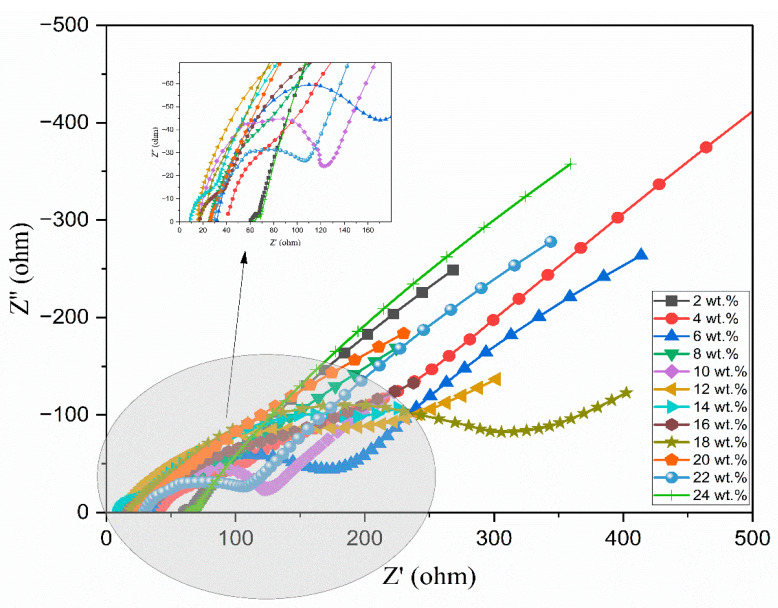
A Nyquist plot of the PEO:NH_4_I with different compositions (wt.%) of TMPI-IL at room temperature.

**Figure 6:**
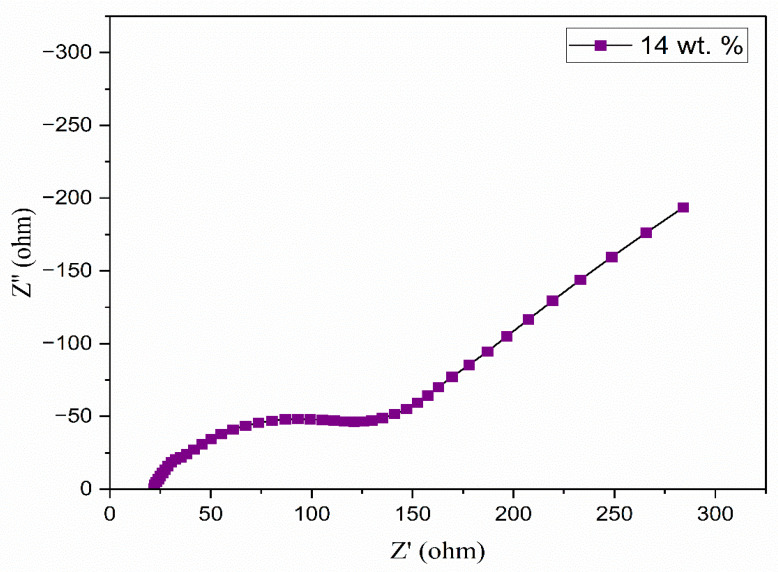
Nyquist plot of 14 wt.% TMPI in PEO:NH_4_I polymer electrolyte.

**Figure 7:**
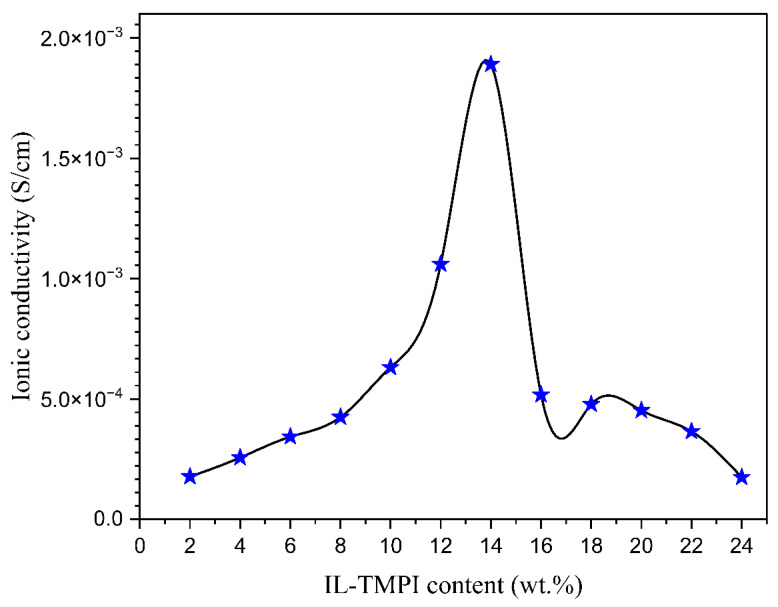
Different compositions of TMPI verse the ionic conductivity achieved.

Ionic liquid was incorporated into the aforementioned polymer electrolyte to improve its conductivity. Figure 5 illustrates the Nyquist diagrams for PEO:NH_4_I containing different compositions (wt.%) of the TMPI ionic liquid in the polymer matrix. A semi-circular arc is observed in all samples, indicating the charge transfer resistance present at the interface between the electrode and the electrolyte. From Figure 5, the ionic conductivity was calculated using Equation (3), and the values are tabulated in Table 3. The ionic conductivity of the optimized polymer electrolyte increases on incorporating up to 14 wt.% of TMPI ionic liquid into the PEO:NH_4_I, as shown in Figure 6 and Figure 7, also shows the corresponding Nyquist diagram. The introduction of TMPI results in a continuous increase in the ionic conductivity but also provides mobile charge carriers that facilitate conduction.

However, after 14 wt.% TMPI, the ionic conductivity began to decrease, which may have been due to the presence of ion pairs associated with charge carriers impeding their mobility within the polymer matrix, as documented in the literature. This restricts the free movement of charge carriers, creating a barrier that ultimately results in reduced conductivity [44].

### 3.6. The Dielectric Analysis

The optimal approach to assessing the increase in the free ion density within the polymer matrix, which leads to enhanced conductivity, is through the evaluation of its dielectric properties. The process of ion transport is significantly influenced by several key factors, and one of these is the dielectric constant (${ɛ}^{*}$) of the polymer electrolyte. A comprehensive understanding of the conductivity mechanisms necessitates an examination of the dielectric properties of the ionically conducting polymer electrolyte. A distinctive characteristic of polymer electrolytes is that the movement of ions transpires without significant long-range displacement of the solvent [45]. The process of ion transport within PEs is complex, involving not only the movement of the ions but also the localized motion of the polymer segments and the transport of ions both within and between polymer chains at coordination sites. The dielectric properties can be determined through calculation by

(4) $${ɛ}^{*}\;=\;{ɛ}^{\prime }\;-\;j{ɛ}^{\prime \prime }$$

where the real part of the complex’s permittivity, often referred to as the dielectric constant, is denoted as ${ɛ}^{\prime }$, while the imaginary part, which is commonly known as the dielectric loss factor, is represented as ${ɛ}^{\prime \prime }$. The dielectric constant generally reflects the polarity of the polymer, as well as the energy accumulated during each cycle of the material when subjected to an alternating electric field. Conversely, the dielectric loss factor signifies the energy dissipated due to the movement of ions. Both the electric constant and the loss factor can be determined by

(5) $$\varepsilon^{\prime }=\frac{Z^{\prime \prime }}{\omega C_{o}(Z^{\prime 2}+Z^{\prime \prime 2})}$$

(6) $$\varepsilon^{\prime \prime }=\frac{Z^{\prime }}{\omega C_{o}(Z^{\prime 2}+Z^{\prime \prime 2})}$$

where $\left(C_{0}=\varepsilon_{0}A/l\right)$ and $\varepsilon_{0}$ represents the permittivity of a vacuum, valued at 8.854 × 10^−12^ Fm^−1^; the contact area between the electrolyte and the blocking electrode is denoted by A, which measures 1.13 cm^2^; and the thickness of the electrolyte films is indicated by l. The angular frequency is represented by ω= 2πf, while $Z^{\prime }$ signifies the real part of the impedance and $Z^{\prime \prime }$ indicates the imaginary part of the impedance.

**Figure 8:**
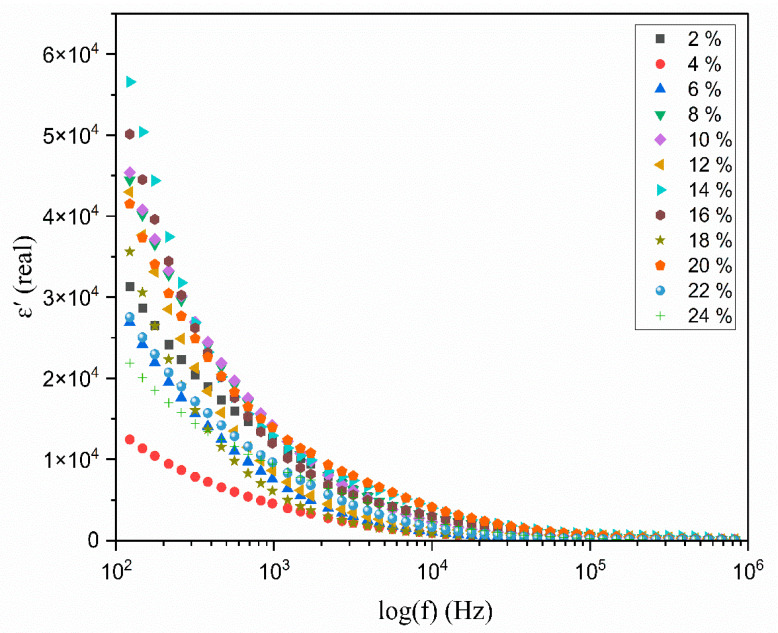
Variation in the dielectric constant $\left({ɛ}^{\prime }\right)$ with the log frequency (Hz) for different wt.% of TMPI in the PEO:NH_4_I polymer electrolyte at room temperature.

**Figure 9:**
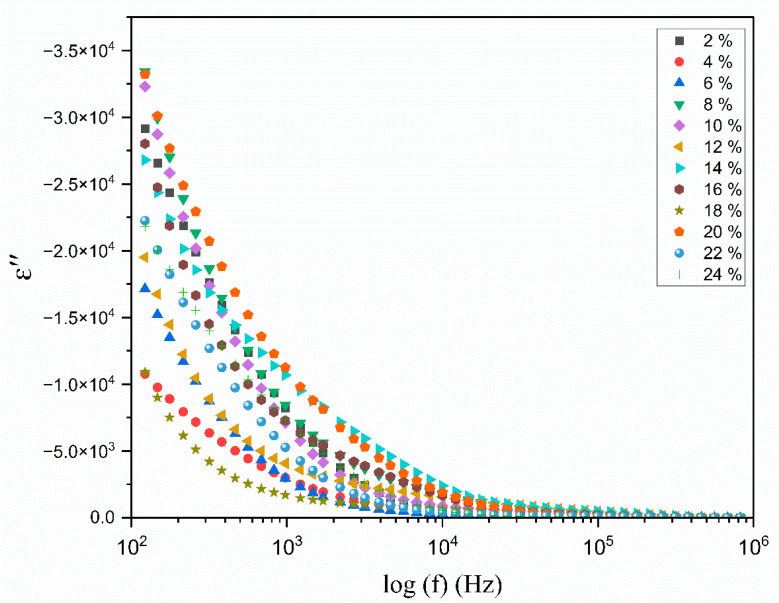
Variation in the dielectric loss $\left({ɛ}^{\prime \prime }\right)$ with the log frequency (Hz) for different wt.% of TMPI in the PEO:NH_4_I polymer electrolyte at room temperature.

**Figure 10:**
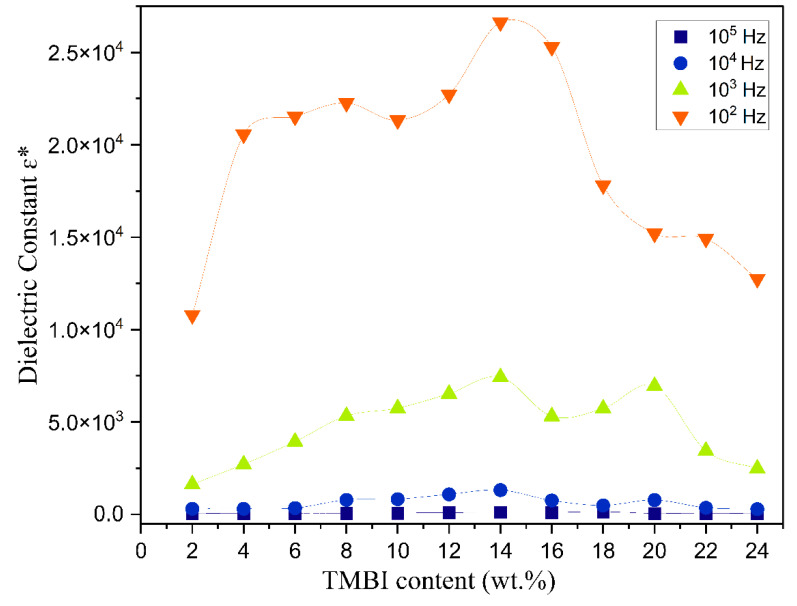
A graph comparing ${ɛ}^{*}$ at various frequencies with different wt.% of TMPI in the PEO:NH_4_I polymer electrolyte.

Figure 8 and Figure 9 illustrate the change in the real and imaginary components of the dielectric constants ($\varepsilon^{\prime }$ and $\varepsilon^{\prime \prime }$) as a function of frequency for the polymer electrolyte and different TMPI compositions (2–24 wt.%) at room temperature. The data presented in both figures indicate that frequency dispersion in $\varepsilon^{\prime }$ and $\varepsilon^{\prime \prime }$ is observed in the low-frequency range, particularly at 10^2^ Hz, where the values are notably high. Subsequently, there is a sharp decline in these values as the frequency increases. The values of $\varepsilon^{\prime }$ and $\varepsilon^{\prime \prime }$ are anticipated to stabilize in the high-frequency region of both spectra; however, the lack of observed steady states is attributed to the restricted frequency range achievable in this experiment. Consequently, only the dispersion component of the dielectric constants is observable at lower frequencies, with the trend in this dispersion’s variation illustrated in the insets in Figure 8 and Figure 9. Overall, the significant rise in $\varepsilon^{\prime }$ and $\varepsilon^{\prime \prime }$ in low-frequency ranges can be attributed to the buildup of space charge carriers at the interface between the electrode and the electrolyte, as well as the alignment of the dipoles from localized molecular polar groups that contribute to polarization. The space charge phenomenon results in the confinement of the charge carriers, as the ions cannot interact with the solid-state blocking electrodes [46]. The value for the 14 wt.% TMPI-doped PEO;NH_4_I exhibits a significantly high dielectric (ɛ*) of 2.66 × 10^4^ at room temperature, as shown in Figure 10. As a result, the polymer electrolyte with 14 wt.% TMPI demonstrates the highest ionic conductivity among all of the polymer electrolytes. This increase in the dielectric constant values can be attributed to the enhanced ionic conductivity of the polymer matrix, which is a result of the uniform distribution of the charge carriers within the polymer matrix. Conversely, there is a significant decline in the high-frequency range across all samples, primarily due to the rapid periodic reversal of the applied electric field. This rapid change limits the time available for the charge carriers to align with the direction of the electric field. Consequently, there is minimal excess diffusion of ions in the field direction, resulting in a reduction in the dielectric constant values. An increase in the number of charge carriers leads to a more pronounced polarization effect and greater energy dissipation. This phenomenon is evident from Figure 10, particularly for the highly conductive 14 wt.% TMPI-doped polymer electrolyte, and demonstrates the highest dielectric constant ($\varepsilon^{\prime }$ and $\varepsilon^{\prime \prime }$) in the lower-frequency range. This enhancement is primarily attributed to the free ions of TMPI, which elevate the charge carrier concentration in the polymer matrices. Consequently, the free mobile ions may associate and form pairs and aggregates, leading to the formation of clusters [47]. This clustering effect subsequently diminishes the electric constant value at a higher TMPI value. The findings from this study align well with the previously discussed ionic conductivity results.

Additionally, the dielectric loss tangent (tan δ) was calculated for each electrolyte to understand the relaxation mechanisms of the electrolyte. The tan δ represents the ratio of energy dissipated to the electric field, referred to as the dissipation factor. The tan δ is derived using Equation (7).

(7) $$tan\;\delta =\frac{\varepsilon^{\prime \prime }}{\varepsilon^{\prime }}$$

**Figure 11:**
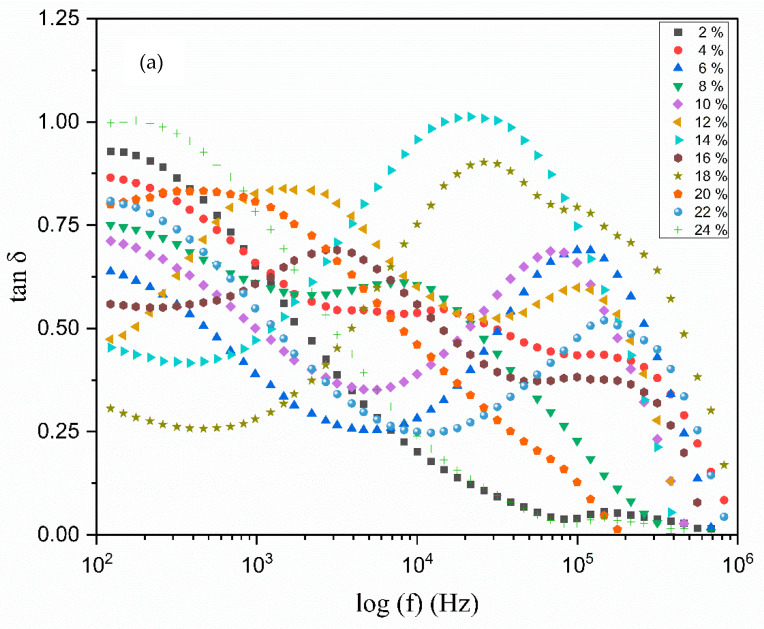

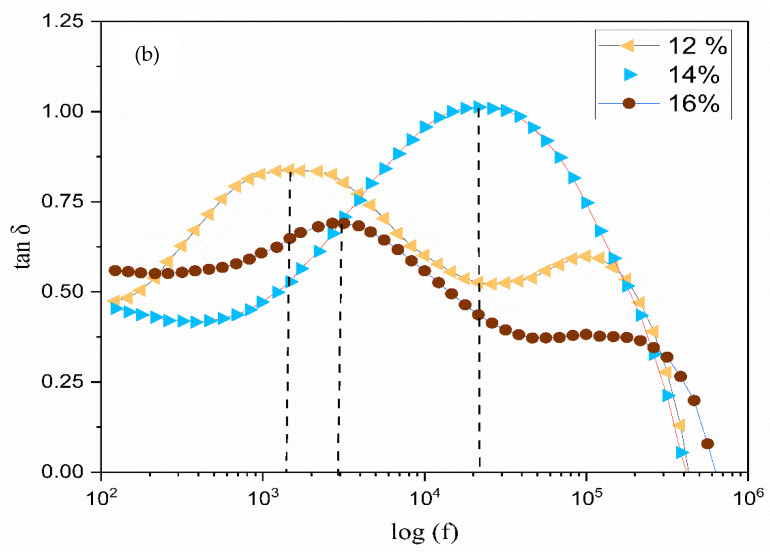
Frequency dependence of tan δ of (**a**) polymer electrolyte on different TMPI contents (**b**), 12 wt.%, 14 wt.%, and 16 wt.%, with curves as examples for finding peak angular frequency.

The relaxation mechanisms of polymer electrolytes are specifically examined through an analysis of the loss tangents peaks, with the dipoles of the polymer electrolyte elucidated in the context of dielectric relaxation. Figure 11a illustrates the relationship between the loss tangent, dielectric relaxation, and frequency for all polymer electrolytes. The peak of the tangent delta is observed to shift towards a higher-frequency range relative to the loss tangent peak, suggesting that the relaxation process is absent in the electrolyte [48]. It is widely recognized that the presence of permanent or induced dipoles contributes to conductivity and the emergence of the dielectric relaxation peak in the graph. The peaks depicted in Figure 11a reflect translational dynamics of ions which are linked to the relaxation of ionic conductivity. As the frequency increases, the tan δ also rises due to the predominance of the ohmic (active) component over the capacitance reactive element [49].

**Figure 12:**
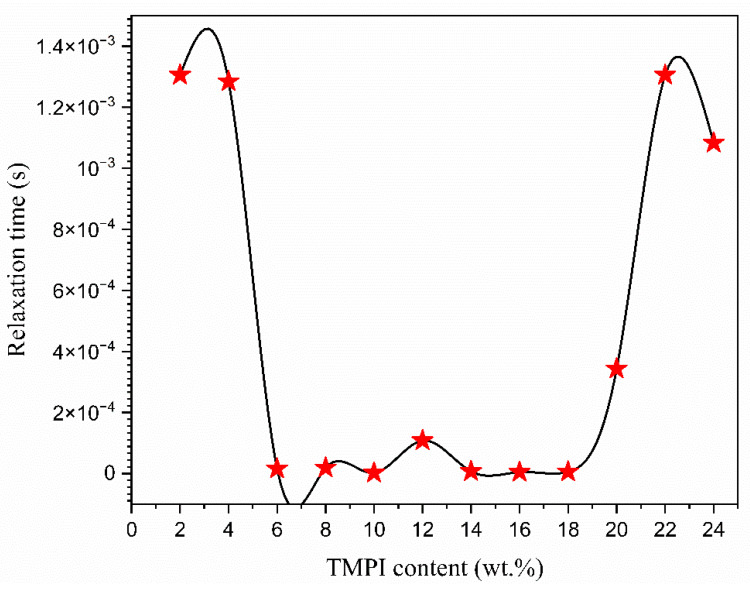
Plot of relaxation times versus different contents of TMPI in PEO:NH_4_I polymer electrolyte.

It is evident that the dielectric loss, represented by tan δ, rises with an increase in frequency, attaining a peak value before subsequently declining with further frequency increments. It is important to highlight that all of the electrolyte films display distinct peaks, which shift towards higher frequencies as the concentration of TMPI increases [50]. The tan δ value at the highest frequency is referred to as the (tanδ_max_) value, which is utilized to ascertain the relaxation peak’s angular frequency (ω), as shown in Figure 11b. Consequently, the relaxation time of the electrolytes is devised from the inverse of peak (1/ω). The values recorded for the relaxation times corresponding to the TMPI concentration are illustrated in Figure 12, and the calculated times are tabulated in Table 4.

The data presented in Table 4 indicates that the 14 wt.% TMPI-doped PEO:NH_4_I polymer electrolyte exhibits the shortest relaxation time of 7.40 × 10^−6^ s. Typically, a reduced relaxation time correlates with an expanded amorphous region, which contributes to greater flexibility of the polymer chains and subsequently improved ionic conductivity [31].

### 3.7. Linear Sweep Voltammetry

**Figure 13:**
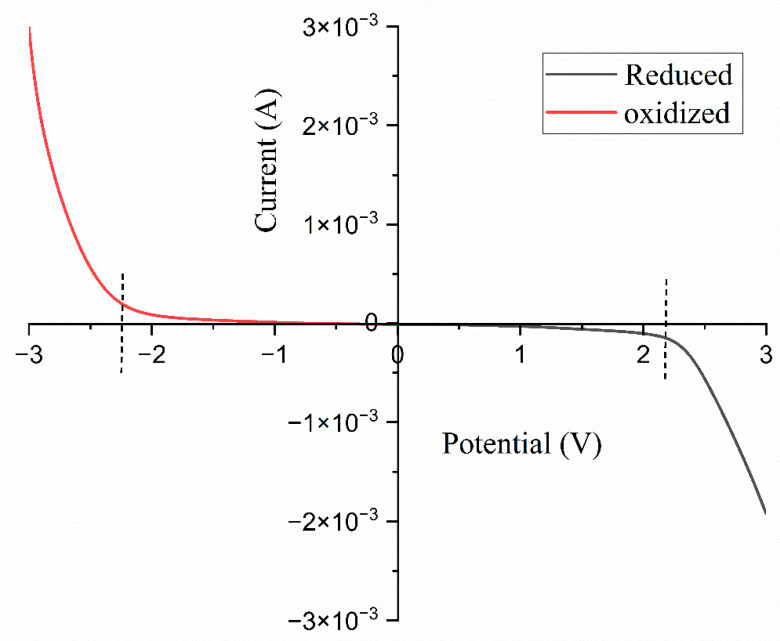
LSV graph for 14 wt.% TMPI-doped polymer electrolyte.

Linear sweep voltammetry (LSV) was employed to assess the potential range of the electrochemical stability window (ESW). The optimized high-conducting polymer electrolyte film was positioned between two stainless steel electrodes and subjected to a voltage range of −3 to 3. Figure 13 presents the LSV graph for the composition of the 14 wt.% TMPI-doped polymer electrolyte: oxidation of the polymer–IL system occurred at −2.35 V, while reduction was observed at 2.23 V, at a sweep rate of 10 mV/s across the electrolyte, aligning with prior studies. The electrochemical stability window for the 14 wt.% TMPI-doped polymer electrolyte was determined to be approx. 4.58 V, thus indicating its suitability for the development of electrochemical devices.

This attribute signifies that the material can function effectively within a specific voltage range without experiencing degradation or adverse reactions. Consequently, this stability renders it a suitable option for the development and enhancement of various electrochemical devices that necessitate a dependable performance under specific voltage conditions [51].

### 3.8. The Ionic Transference Number

**Figure 14:**
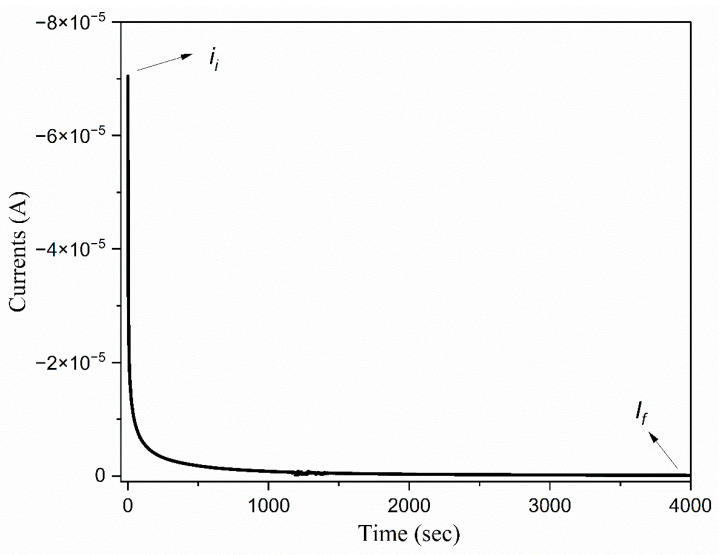
DC polarization curve of TMPI-doped PEO:NH_4_I polymer electrolyte.

The total ionic transference number is calculated using the DC polarization method. This technique is utilized to evaluate the effectiveness of the synthesized electrolyte films in promoting ionic conduction. The ionic transference number ($t_{ion}$) is given by the following, Equation (7):

(8) $$t_{ion}=\frac{I_{i}\;-\;I_{f}}{I_{i}}$$

where $I_{i}$ represents the initial current, and $I_{f}$ denotes the final stabilized current. Figure 14 illustrates the direct current (DC) polarization curves of electrolyte films containing 14 wt.% concentrations of TMPI. The ion transference number for the 14 wt.% TMPI-doped PEO:NH_4_I polymer electrolyte is measured at 0.98, indicating that the system exhibits ion conductivity. It is evident that electrolyte systems initially display a significant polarization current, which subsequently declines sharply before stabilizing after prolonged polarization. The initial current is due to the result of the movement of both ions and electrons, representing the total current. In contrast, the final stabilized current is solely attributed to the electron flow [52]. When the sandwiched test model is subjected to a DC voltage of 0.5 V, the polarization current decreases over time, ultimately reaching a steady state due to the establishment of a chemical potential gradient within which the electrons traverse freely, thereby confirming that ionic conduction predominates in these electrolyte films. The electrode obstructs the movement of the ions, while the electrons are able to pass through without restriction. This observation supports the conclusion that ionic conduction is the primary mechanism in this electrolyte system [53].

It is important to note that the polymer electrolyte comprises phosphonium cations (C_13_H_30_P^+^) and iodide anions (I^−^); however, the predominant factor influencing the ionic conduction is attributed to the iodide anionic species, which is smaller in size and more mobile in the polymer’s segmental motion, as the phosphonium cations are bulky. As the proportion of ionic liquid in the composition increases, the concentration of I^−^ rises, leading to an eventual peak in the ion transference number. Nevertheless, an excessive concentration of ionic liquid may lead to the formation of ion pairs or ion aggregates and create a blocking effect within the space charge region. This significantly impedes the mobility of the charge carriers, consequently reducing the ion transference number [54].

## 4. The Performance of Dye-Sensitized Solar Cells 

**Figure 15:**
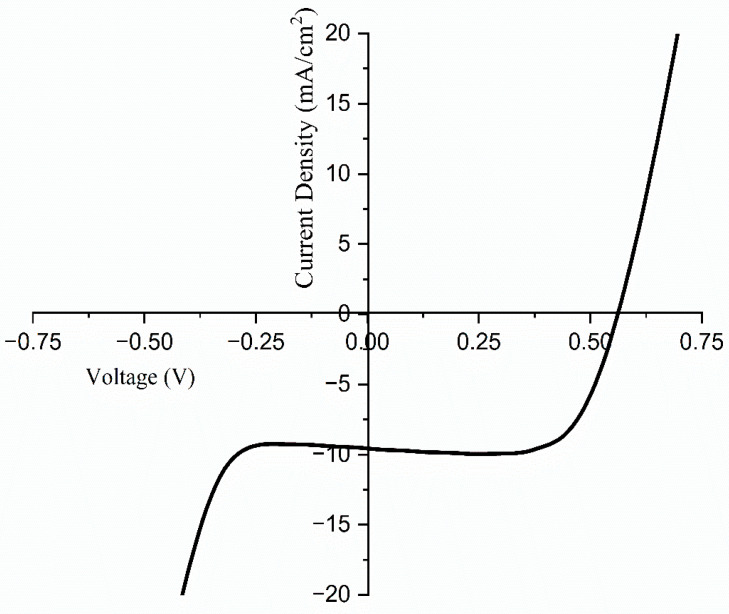
Current density–voltage curves obtained for 14 wt.% TMPI-doped PEO:NH_4_I polymer electrolyte under one sun conditions.

**Figure 16:**
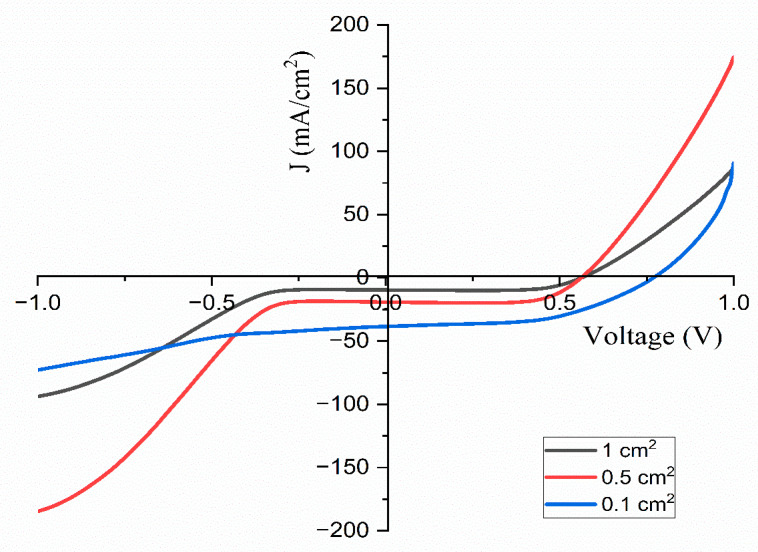
Current density–voltage curves obtained for 14 wt.% TMPI-doped PEO:NH_4_I polymer electrolyte under one sun conditions for different areas of contact.

The current density–voltage (J-V) characteristics were evaluated for solar cells constructed in the configuration of FTO/blocking layer/TiO_2_/dye/Pt/FTO. A solar simulator, designated as the model SS-F5-3A and manufactured by Enlitech in Taiwan, was employed to assess the performance of the developed DSSCs. These results are illustrated in Figure 15 and detailed in Table 5. The J-V curves for solar cells with different areas of contact with the optimized TMPI-doped polymer electrolyte under one sun conditions are presented in Figure 16. The performance of the solar cells is determined using Equation (10).

(9) $$\eta =\frac{J_{sc}V_{oc}FF}{P_{in}}$$

In this context, $J_{sc}$ refers to the short-circuit current density, $V_{oc}$ stands for the open circuit voltage, and FF represents the fill factor.

(10) $$FF=\frac{P_{max}}{J_{sc}V_{oc}}$$

The fill factor itself is calculated using Equation (11), which takes into account the maximum power output of the solar cell relative to its theoretical output. Finally, the maximum power output can be achieved using the formula [$P_{max}=V_{max}\times J_{max}$], while the overall efficiency of the DSSC is denoted by (η,%). Current is produced when the negative and positive electrodes of the cell are linked in a short circuit at zero millivolts [12].

The introduction of ionic liquid into the PEO:NH_4_I polymer electrolyte markedly improves the photovoltaic performance of the dye-sensitized solar cell (DSSC). This enhancement is reflected in the increased parameters and the overall photoelectric energy conversion efficiency, which suggests superior charge dynamics and improved electron transport within the doped electrolyte, resulting in more effective solar energy conversion [55]. The data presented in Table 5 indicate that the constructed DSSCs exhibit efficiencies varying from 3.83% to 15.14% under the standard one sun illumination, depending on the effective area.

**Figure 17:**
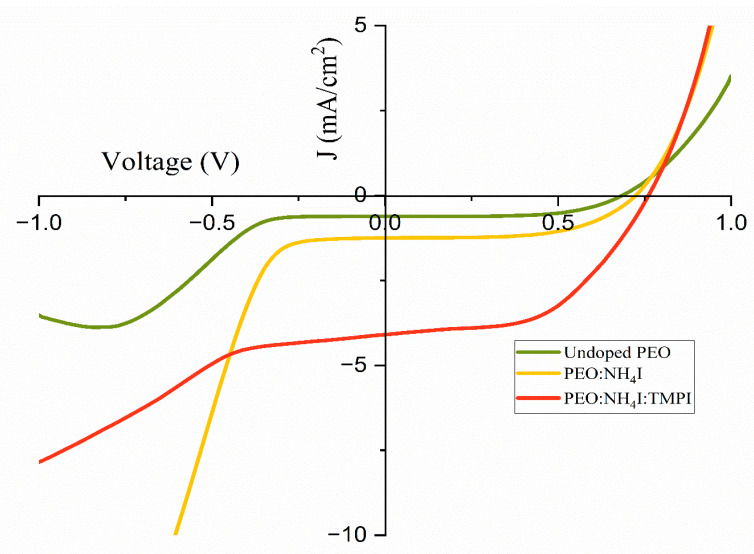
Comparison of J-V curves of undoped PEO, PEO:NH_4_I, and PEO:NH_4_I:TMPI polymer electrolyte.

From Figure 17 and Table 6, the results indicate that the DSSC utilizing the TMPI-ionic-liquid-doped polymer electrolyte outperforms the DSSCs that employs the PEO:NH_4_I complex and the undoped PEO polymer electrolyte. These findings indicate significant improvements in both V_oc_ and J_sc_ upon the modification of the polymer electrolyte with the TMPI ionic liquid. This observation underscores the distinct relationship between V_oc_ and J_sc_ after the introduction of TMPI, implying that the enhancement in the ionic conductivity contributes to this association.

**Figure 18:**
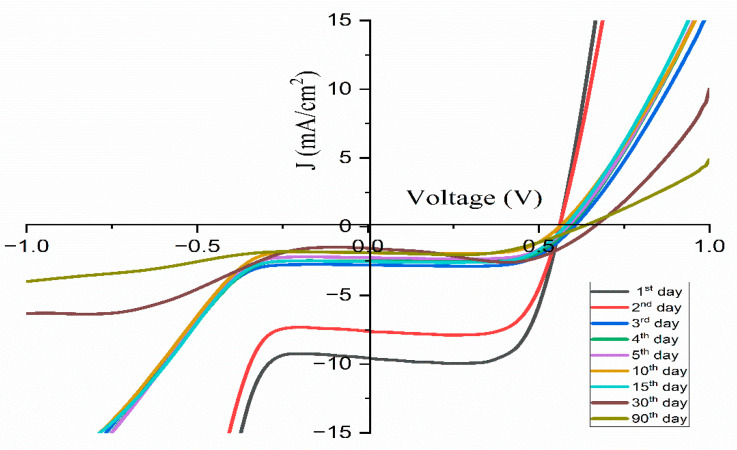
Variation in J-V curves for DSSCs under one sun conditions in different time periods.

**Figure 19:**
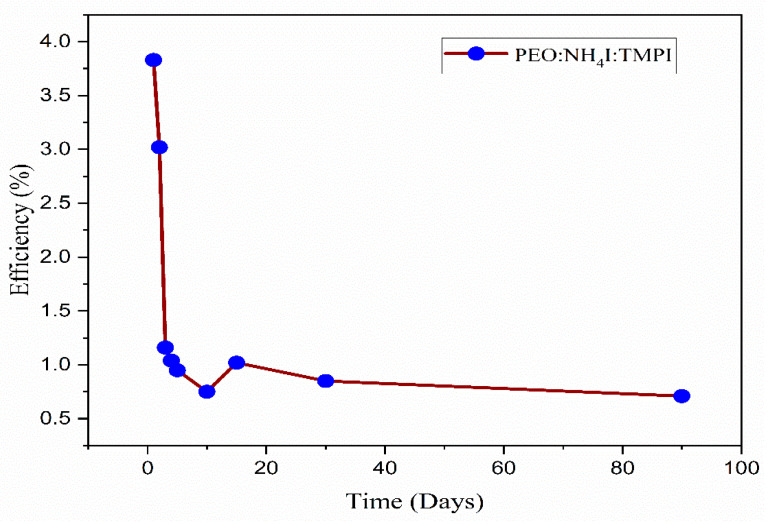
Curves of the efficiency of the DSSC over time.

**Figure 20:**
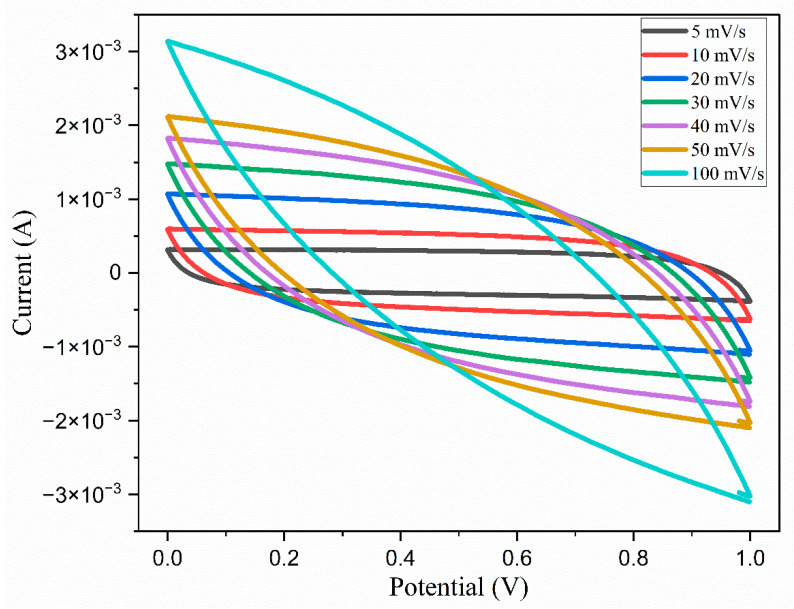
Cyclic voltammograms of the EDLC obtained at different scan rates.

To evaluate the standard application of these cells, the JSC and VOC parameters were continuously monitored for a period of 90 days as shown in Figure 18. The findings are summarized in Table 7, which refers to the DSSC using a polymer electrolyte composed of 14 wt.% TMPI. The data indicate a consistent performance up to the fourth day, as shown in Figure 19. Consequently, the DSSC with the TMPI-doped polymer electrolyte demonstrates exceptional long-term stability, as shown in Figure 20. The observed decrease in efficiency after 10 days may be due to volatilization and leakage, which occur during prolonged operation and negatively affect the performance of DSSCs. However, by day 30, a slight recovery in efficiency was recorded, which may have been due to stabilization of the polymer electrolyte’s morphology or an improvement in ionic conduction after the initial relaxation and recombination stage [56]. However, the efficiency of the DSSC using doped TMPI shows an improvement, confirming that TMPI not only improves the conductivity but also increases the efficiency of the DSSC.

## 5. The Electric Double-Layer Capacitor’s Performance

### 5.1. Cyclic Voltammetry

Cyclic voltammograms were recorded over a potential range of 0 to 1 V using different sweep rates, as illustrated in Figure 20. In cases where the capacitance remains stable despite potential variations, the resulting CV curves exhibit a distinctive rectangular profile. However, when the capacitance is affected by the potential, the voltammograms exhibit a consistent rectangular shape at all sweep rates. This indicates potential-independent capacitance behavior. This shape also implies rapid ionic switching at the interfaces between the electrode and the electrolyte, as well as the favorable capacitive characteristics of the electrodes. From the CV curves, the specific capacitance can be calculated from the following, Equation (12):

(11) $$C_{s}=\frac{1}{mv∆V}\int IdV$$

where v represents the scan rate; V denotes the cell voltage; and m indicates the mass of the active material, specifically activated carbon, per electrode, measured in grams. The specific capacitance values calculated are tabulated in Table 8.

The data presented in Table 8 indicates that at a 5 mV/s scan rate, a specific capacitance of 205.6 F/g is exhibited. This capacitance is influenced by various factors, including the resistance to ion transport and the rate of ion diffusion and migration, as well as the diffusion length. It has been observed that the specific capacitance of both cells diminishes progressively as the scan rates increase, resulting in a loss of capacitance from the initial value for the EDLC. As the scan rates rises, there is a corresponding increase in the energy loss, accompanied by a reduction in the charge retained on the electrode’s surface, ultimately leading to a decline in the specific capacitance [57].

### 5.2. Low-Frequency Impedance Capacitance

**Figure 21:**
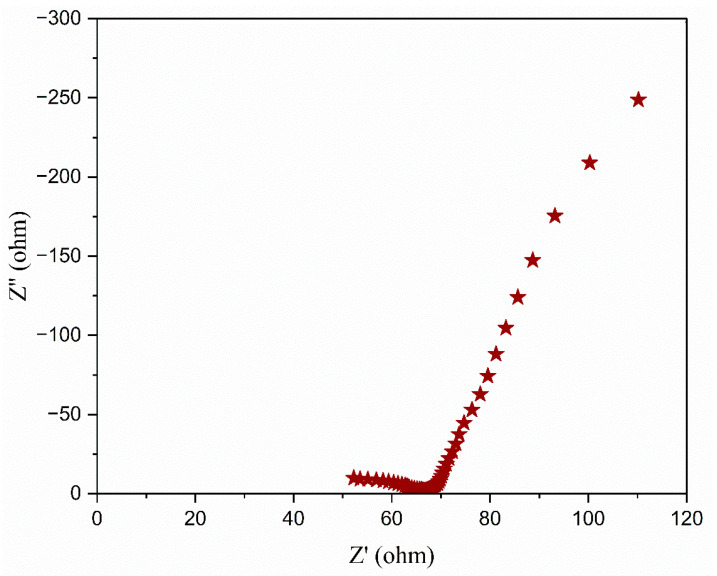
Low-frequency impedance curve of fabricated EDLC with TMPI-doped polymer electrolyte.

The low-frequency impedance was assessed at the CH workstation to evaluate the electrical characteristics of the developed EDLC devices. Characteristics of an initial frequency of 10^−2^ Hz and a maximum frequency of 10^5^ Hz were required. Figure 21 illustrates the low-frequency impedance graph for the EDLC. The capacitance was calculated by applying Equation (12).

(12) CLF=1−2πωZ″

Here, ω denotes the frequency, $Z^{\prime \prime }$ represents the imaginary component of the impedance plot, and $C_{LF}$ dignifies the specific capacitance at low frequency.

The graph clearly shows a linear relationship, indicating bulk resistance in the low-frequency region, specifically in the $Z^{\prime \prime }$ area (on the *y*-axis). According to Equation (12), the calculated capacitance reached 73.69 F at the peak value in the $Z^{\prime }$ area (on the *x*-axis). The capacitance value derived from low-impedance spectroscopy can be compared with the value obtained from the cyclic voltammetry profile at 50 mV/s (Table 8). The capacitance values from both methodologies are nearly identical, as they reflect ion transport dynamics that are close to equilibrium [58].

## 6. Conclusions

Polyethylene oxide (PEO)-based polymer electrolytes incorporating ionic liquid (TMPI) have been efficiently synthesized. This investigation delves into the electrical characteristics of the TMPI-doped polymer electrolytes, which include an ammonium iodide system (from previous work). The impedance analysis of the TMPI-doped polymer electrolytes revealed a decrease in the resistance (R_b_) with an increasing TMPI composition, reaching an optimal conductivity of 1.89 × 10^−2^ S/cm at 14 wt.% TMPI, indicating an improvement in conductivity with the incorporation of TMPI. Dielectric studies provide insight into the behavior of the PEO-based polymer with TMPI, indicating a decrease in high-frequency performance due to the presence of charge carriers in the polymer matrix. Investigations into the characteristics of ionic transport indicate that conductivity is influenced by ionic mobility, the diffusion coefficient of the charge carriers, and their density. X-ray diffraction results confirm the amorphous nature of the polymer electrolyte, which facilitates ionic distribution and improves the conductivity. Fourier transform infrared spectroscopy reveals that the peaks of the TMPI-doped polymer electrolyte exhibit strong interactions with PEO and NH_4_I. The differential scanning calorimetry curves demonstrate that the incorporation of TMPI into the PEO polymer electrolyte lowers both the melting transition temperature and the crystallinity of the system. Meanwhile, the enhancement in the efficiency and specific capacitance of DSSCs and EDLCs using the 14 wt.% TMPI-doped polymer electrolyte is modest but shows some improvement, reinforcing the idea that TMPI improves the conductivity and marginally enhances the electrochemical performance of energy storage devices. In summary, the proposed TMPI-doped polymer electrolyte system shows significant potential for application as an electrolyte material in DSSCs and EDLCs.

## Figures and Tables

**Table 2:** Various thermal parameters from DSC curves for pure PEO, PEO: NH_4_I, and PEO: NH_4_I:TMPI.

Composition of Polymer Electrolyte	T_onset_ (°C)	T_offset_ (°C)	${∆T}_{m}$	∆H_m_ (J/g)	$\chi_{c}$ (%)
Pure PEO	56.35	80.23	23.77	155.45	72.74
PEO:NH_4_I	55.04	78.24	23.10	120.46	56.37
PEO:NH_4_I:TMPI	53.48	76.72	23.14	71.96	33.67

**Table 3:** The ionic conductivity of pure PEO, PEO:NH_4_I, and different compositions (wt.%) of TMPI in the polymer electrolyte.

Composition of PEO, NH_4_I, TMPI (wt.%)			Ionic Conductivity (S/cm)
PEO	NH_4_I,	TMPI	
100	0	0	2.96 × 10^−8^
100	8	0	2.05 × 10^−6^
100	8	2	1.77 × 10^−4^
100	8	4	2.56 × 10^−4^
100	8	6	3.43 × 10^−4^
100	8	8	4.24 × 10^−4^
100	8	10	6.31 × 10^−4^
100	8	12	1.06 × 10^−3^
100	8	14	1.89 × 10^−3^
100	8	16	5.17 × 10^−4^
100	8	18	4.78 × 10^−4^
100	8	20	4.52 × 10^−4^
100	8	22	3.65 × 10^−4^
100	8	24	1.74 × 10^−4^

**Table 4:** Relaxation times of different compositions of TMPI-doped polymer electrolytes.

TMPI Content (wt.%)	Relaxation Frequency (Hz)	Peak Angular Frequency (rad/s)	Relaxation Time (s)
2	122	766.16	1.31 × 10^−3^
4	124	778.72	1.28 × 10^−3^
6	9960	62,548.8	1.59 × 10^−5^
8	8300	52,124	1.91 × 10^−5^
10	68,100	427,668	2.33 × 10^−6^
12	14,700	19,231.6	1.08 × 10^−4^
14	21,500	135,020	7.40 × 10^−6^
16	31,700	199,076	5.02 × 10^−6^
18	26,100	163,908	6.10 × 10^−6^
20	464	2913.92	3.34 × 10^−4^
22	122	766.16	1.13× 10^−3^
24	147	923.16	1.80 × 10^−3^

**Table 5:** DSSC parameters evaluated for PEO, PEO:NH_4_I, and PEO:NH_4_I:TMPI polymer electrolyte.

Area $\left({\mathrm{cm}}^{2}\right)$	$I_{sc}\;(\mathrm{mA})$	$V_{oc}\;(V)$	Jsc $\left(\mathrm{mA}/{\mathrm{cm}}^{2}\right)$	Fill Factor (%)	Efficiency (%)
1	1.91	0.56	9.56	71.42	3.83
0.5	1.91	0.56	19.16	71.03	7.64
0.1	1.90	0.56	38.44	51.22	15.14

**Table 6:** DSSC parameters of undoped PEO, PEO:NH_4_I, and PEO:NH_4_I:TMPI polymer electrolyte.

Polymer Electrolyte	$I_{sc}\;(\mathrm{mA})$	$V_{oc}\;(V)$	Jsc $\left(\mathrm{mA}/{\mathrm{cm}}^{2}\right)$	Fill Factor (%)	Efficiency (%)
Undoped PEO	0.61	0.68	0.61	61.89	0.26
PEO:NH_4_I	0.31	0.72	1.24	58.51	0.52
PEO:NH_4_I:TMPI	1.91	0.56	9.56	71.42	3.83

**Table 7:** Changes in DSSC parameters for TMPI-doped PEO:NH_4_I over different time periods.

$I_{sc}\;(\mathrm{mA})$	$V_{oc}\;(V)$	Jsc $\left(\mathrm{mA}/{\mathrm{cm}}^{2}\right)$	FillFactor (%)	Efficiency (%)	Time (days)
1.91	0.56	9.56	71.42	3.83	1
1.90	0.56	7.61	70.91	3.02	2
2.78	0.60	2.78	69.14	1.16	3
2.49	0.59	2.49	70.42	1.04	4
2.25	0.58	2.25	72.18	0.95	5
1.89	0.56	1.89	71.19	0.75	10
2.54	0.57	2.54	70.07	1.02	15
1.57	0.66	1.57	81.82	0.85	30
1.88	0.62	1.88	60.15	0.71	90

**Table 8:** Specific capacitance values of the fabricated EDLC with the TMPI-doped polymer electrolyte at different scan rates.

Scan Rate	Specific Capacitance (F/g)
5	205.6
10	171.5
20	134
30	109.7
40	91.37
50	76.89
100	13.90

## Data Availability

The data underpinning the results of this study can be obtained from the corresponding author upon reasonable request.
